# Non‐invasive phrenic nerve stimulation to avoid ventilator‐induced diaphragm dysfunction in critical care

**DOI:** 10.1111/aor.14244

**Published:** 2022-04-12

**Authors:** Conor Keogh, Francisco Saavedra, Sebastian Dubo, Pablo Aqueveque, Paulina Ortega, Britam Gomez, Enrique Germany, Daniela Pinto, Rodrigo Osorio, Francisco Pastene, Adrian Poulton, Jonathan Jarvis, Brian Andrews, James J. FitzGerald

**Affiliations:** ^1^ Nuffield Department of Surgical Sciences University of Oxford Oxford UK; ^2^ Electrical Engineering Department Universidad de Concepcion Concepción Chile; ^3^ Kinesiology Department Universidad de Concepcion Concepción Chile; ^4^ School of Sport and Exercise Science Liverpool John Moores University Liverpool UK

**Keywords:** critical care, electrical stimulation, phrenic nerve, ventilator‐induced diaphragm dysfunction

## Abstract

**Background:**

Diaphragm muscle atrophy during mechanical ventilation begins within 24 h and progresses rapidly with significant clinical consequences. Electrical stimulation of the phrenic nerves using invasive electrodes has shown promise in maintaining diaphragm condition by inducing intermittent diaphragm muscle contraction. However, the widespread application of these methods may be limited by their risks as well as the technical and environmental requirements of placement and care. Non‐invasive stimulation would offer a valuable alternative method to maintain diaphragm health while overcoming these limitations.

**Methods:**

We applied non‐invasive electrical stimulation to the phrenic nerve in the neck in healthy volunteers. Respiratory pressure and flow, diaphragm electromyography and mechanomyography, and ultrasound visualization were used to assess the diaphragmatic response to stimulation. The electrode positions and stimulation parameters were systematically varied in order to investigate the influence of these parameters on the ability to induce diaphragm contraction with non‐invasive stimulation.

**Results:**

We demonstrate that non‐invasive capture of the phrenic nerve is feasible using surface electrodes without the application of pressure, and characterize the stimulation parameters required to achieve therapeutic diaphragm contractions in healthy volunteers. We show that an optimal electrode position for phrenic nerve capture can be identified and that this position does not vary as head orientation is changed. The stimulation parameters required to produce a diaphragm response at this site are characterized and we show that burst stimulation above the activation threshold reliably produces diaphragm contractions sufficient to drive an inspired volume of over 600 ml, indicating the ability to produce significant diaphragmatic work using non‐invasive stimulation.

**Conclusion:**

This opens the possibility of non‐invasive systems, requiring minimal specialist skills to set up, for maintaining diaphragm function in the intensive care setting.

## Graphical abstract

We demonstrate that the diaphragm can be exercised using non‐invasive electrical stimulation of the phrenic nerve and characterize the stimulation parameters required to achieve this. This opens the possibility of non‐invasive systems for maintaining diaphragm function in ventilated patients in an intensive care setting.

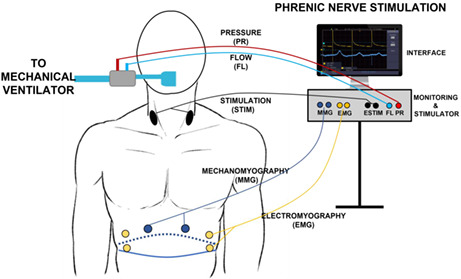

## 1 INTRODUCTION

Mechanical ventilation is an essential intervention in critical care medicine. However, disuse of the diaphragm during ventilation can rapidly result in severe dysfunction, with evidence of significant diaphragm weakening after as little as 24 h.^1^ This “ventilator‐induced diaphragm dysfunction” (VIDD) is associated with difficulty weaning from respiratory support, and the resulting prolonged periods of ventilation may adversely affect clinical outcomes.^2^

Stimulation of the phrenic nerves to induce diaphragm contraction is a promising means of maintaining diaphragm condition during mechanical ventilation. It is the *complete* lack of activity in skeletal muscle that leads to atrophy, and only a small amount of exercise (~200 contractions per day) is needed to prevent it.^3^, ^4^ Phrenic nerve stimulation aims to reduce or reverse the atrophy produced by diaphragm inactivity by “exercising” the diaphragm.

There are currently multiple invasive techniques for achieving stimulator‐induced diaphragm activation.^5^ These can be broadly classified as transvenous methods, where the phrenic nerve is stimulated using electrodes inserted via the internal jugular or subclavian vein,^6^, ^7^ and diaphragm pacing, where electrodes are surgically implanted directly in the diaphragm.^8^, ^9^ Transvenous pacing has been shown to maintain diaphragm conditions in animals,^10^ while both implanted^11^ and percutaneous^12^ stimulators have shown potential value in humans. However, invasive methods of phrenic nerve stimulation have a number of disadvantages that may limit their widespread application. Specific technical skills are required for electrode insertion: for example, transvenous pacing requires placement of jugular or subclavian vein catheters and diaphragm pacing requires surgical implantation or percutaneous access. These carry risks of mechanical complication during the insertion procedure (for example, central venous access carries risks of arterial puncture, hematoma, or pneumothorax) and further potential complications thereafter (thrombosis and catheter infection),^13^ although the specific risks vary with the insertion route chosen.

Non‐invasive stimulation of the phrenic nerves would offer a means to maintain diaphragm condition during mechanical ventilation while avoiding the risks and technical demands of more invasive approaches. The ability to induce diaphragm contraction using surface electrodes has been demonstrated before, however, these approaches rely on frames,^14^ collars,^15^, ^16^ and hand‐held probes^17^ to apply pressure in order to improve coupling by reducing the distance between the electrode and the nerve. The need for pressure renders these methods unsuitable for extended use in an intensive care setting because of the risk of generating pressure sores, as does their inability to account for changes in the position of the phrenic nerve relative to the skin as the position of the head is moved during routine care. Additionally, one cause of lengthy periods of ventilation is a traumatic brain injury, and in that situation, the application of any external pressure in the region of the jugular veins is absolutely contraindicated due to the danger of raising intracranial pressure by creating increased resistance to venous return from the brain.

Our group, therefore, aimed to develop a non‐invasive phrenic nerve stimulation system suitable to maintain diaphragm muscle function in ventilated patients in an intensive care setting. This requires reliable ventilator‐synchronized diaphragm contraction using surface electrodes without the application of pressure.

Here, we demonstrate that non‐invasive capture of the phrenic nerve is possible and characterize the parameters required to achieve meaningful diaphragm contraction in healthy volunteers. This opens the possibility for non‐invasive methods to maintain diaphragm function in ventilated patients, with the potential to reduce time spent on a ventilator and improve clinical outcomes.

## 2 METHODS

Healthy volunteers with no respiratory or neurological pathologies were recruited. The study was approved by the University of Oxford Central University Research Ethics Committee (approval reference R73898/RE001) and by the Ethics Committee at the Universidad de Concepción, Chile (CEBB 714–2020).

### 2.1 Electrode design

**FIGURE 1:**
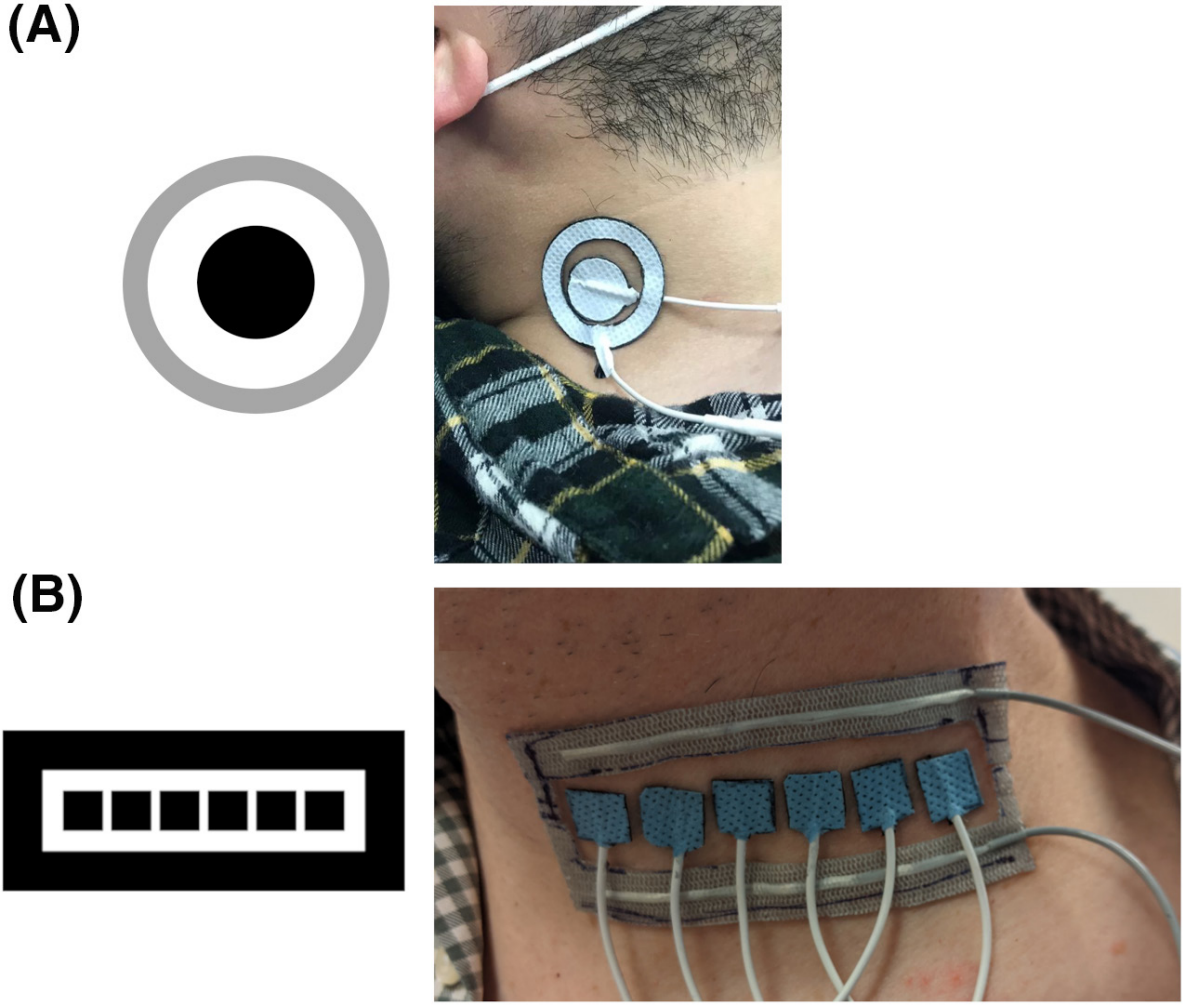
Electrode design. (A) Schematic and photograph of concentric electrode for initial testing. A 20 mm diameter cathode is surrounded by a ring‐shaped anode with an inner diameter of 30 mm and an outer diameter of 40 mm. (B) Schematic and photograph of linear array used for parameter optimization. Six 1 cm × 1 cm cathodes, separated by 2 mm intervals, are surrounded by an anode

Custom electrode geometries were created by modification of commercial (Axelgaard) electrodes. Electrode geometries are shown in Figure 1.

For the initial assessment of the efficacy of non‐invasive phrenic nerve stimulation, concentric ring electrodes, with an inner cathode surrounded by a ring‐shaped anode, were used to constrain the area of activation (Figure 1A). Circular cathodes were fabricated with a 20 mm diameter. Ring‐shaped anodes were fabricated with an inner diameter of 30 mm and an outer diameter of 40 mm.

In order to characterize the impact of electrode position on diaphragm response, linear electrode arrays were fabricated (Figure 1B). Six 1 cm × 1 cm cathodes separated by 2 mm gaps were placed in a linear array on the neck, surrounded by a 1‐cm wide anode strip (the inner edge of the anode was 5 mm from the edges of the cathodes). with a typical electrode impedance of 1470 ± 148 Ω. The medial end of the electrode array was placed on the midline with the cathodes at the level of the cricoid cartilage.

### 2.2 Stimulation

**FIGURE 2:**
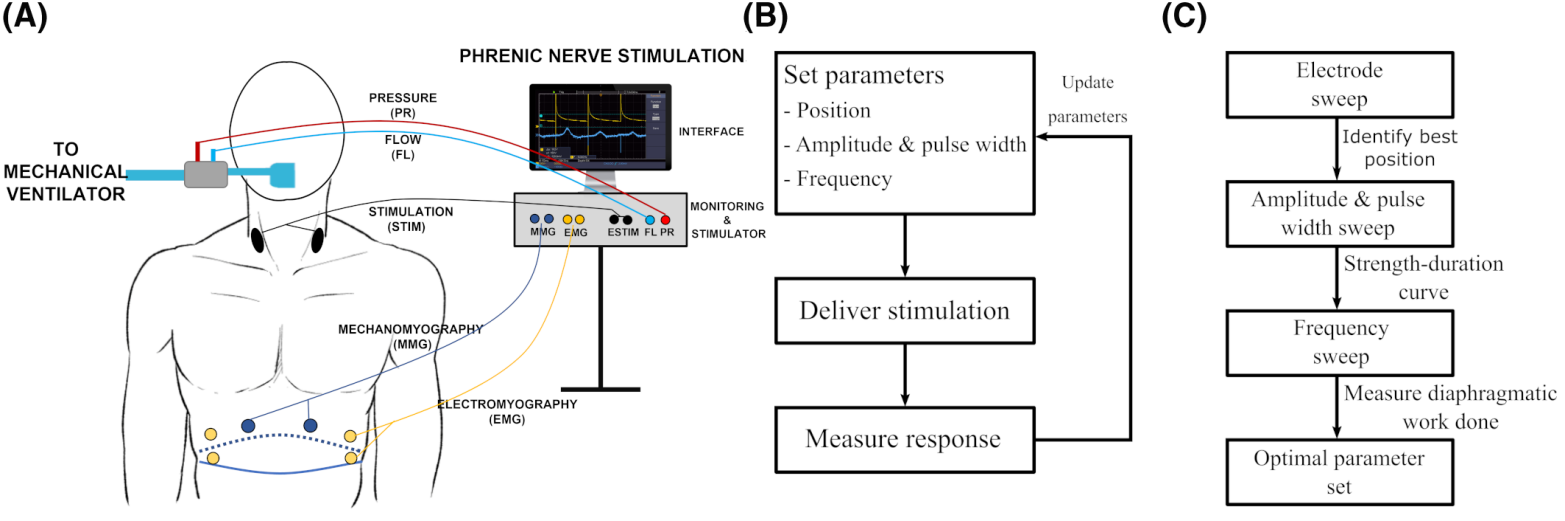
System set up. (A) Schematic stimulation and monitoring equipment. Stimulation electrodes were placed on the neck bilaterally. Respiratory pressure and flow were measured in line with the mechanical ventilator. EMG recordings were taken from bipolar electrodes in the sixth and eighth intercostal spaces bilaterally. MMG was recorded using sensors placed just medial to the EMG electrodes. Stimulation and recording were controlled from a common interface. (B) Flowchart of parameter optimization protocol. Electrode position, amplitude, pulse width, and stimulation frequency were set and delivered via electrodes overlying the phrenic nerve. The response to stimulation was measured and the required parameters were updated according to the response elicited. (C) Flowchart of overall parameter optimization. All electrode positions were assessed. The best position was used for testing the amplitude required to elicit a response at a range of pulse widths. The strength‐duration curve was used to determine pulse train parameters. Pulse trains with varying frequencies were then applied and the work done by the diaphragm measured

A custom stimulation and monitoring system were developed for the initial evaluation of the effect of non‐invasive phrenic nerve stimulation (Figure 2A). A two‐channel stimulator was designed to deliver constant current monophasic stimulation to the phrenic nerve bilaterally. Each independently controlled channel allowed delivery of stimulation pulses from 10 μs to 400 μs with a resolution of 10 μs and amplitudes from 1 mA to 150 mA (assuming a 2 kΩ load, i.e., a compliance voltage of 300 V) with a resolution of 1 mA. Pulse trains could be generated with frequencies from 1 Hz to 30 Hz with a resolution of 1 Hz.

To allow even greater control of stimulation parameters and electrode selection, a detailed assessment of the parameters required to induce diaphragm contraction was performed using a Digitimer DS8R current‐regulated stimulator with a compliance voltage of 400 V. Biphasic, charge‐balanced, symmetric waveforms were used throughout all testing. Each of the six electrodes in the linear array was attached to a Digitimer D188 electrode selector to allow stimulation at individual electrode sites.

### 2.3 Initial assessment

Evaluation of the effect of non‐invasive phrenic nerve stimulation on the diaphragm was performed by stimulating the nerve bilaterally while performing multi‐modal monitoring (Figure 2A).

Participants were placed in a supine position. Blood pressure, heart rate, respiratory rate, and oxygen saturation monitors were installed to allow monitoring during stimulation. The diaphragm electromyogram (EMG) was recorded using bipolar electrode pairs in the sixth and eighth intercostal spaces bilaterally to assess the electrical response to stimulation. The use of a bipolar electrode arrangement at a significant distance from the stimulating electrodes on the neck allowed diaphragm EMG to be measured while avoiding significant contamination with stimulation artifacts.

Mechanomyography (MMG) was performed using accelerometers placed just medial to the EMG electrodes bilaterally to evaluate the mechanical response to stimulation. Participants were connected to a non‐invasive mechanical ventilator (Flight 60 ventilator, Flight Medical Innovation) in continuous mandatory (assist‐control) ventilation mode. This mode allowed patient‐triggered breaths on detection of an inspiratory effort while reverting to ventilator‐triggered breaths at a fixed rate if necessary. This allowed the ventilator to be activated by inspirations generated by stimulation in order to provide pressure support to induced inspirations while enforcing a minimum number of guaranteed breaths per minute if there is an insufficient spontaneous effort to trigger respiration. This allowed the interaction between stimulation‐induced diaphragm contractions and mechanical ventilation to be investigated, including the potential risk of inducing patient‐ventilator asynchronies due to stimulation. Respiratory pressure and flow were measured in line with the ventilator to assess the respiratory response. Diaphragm ultrasound was used to provide direct visualization of the response to stimulation.

A calibration procedure was performed to define the electrode position and stimulation parameters used for an initial assessment. A stimulator probe was used to manually apply stimulation to the area just posterior to the sternocleidomastoid muscle at the level of the cricoid cartilage. Diaphragm response was evaluated using the multi‐modal monitoring setup. This was repeated until the area that elicited the maximal diaphragm response, defined as the location that produced the largest area under the curve on pressure and flow waveforms following stimulation with confirmation of diaphragm contraction on EMG, MMG, and direct visualization was identified bilaterally. Concentric electrodes were then placed in these locations and the stimulation parameters were tuned based on the diaphragm response seen during monitoring.

Following identification of the required electrode position, stimulation amplitude, pulse width, and frequency, pulse trains of 1.1 s with an inter‐pulse interval of 2.2 s were delivered. The ventilator was triggered by stimulation‐induced inspiration, corresponding to a 1:2 inspiration: expiration ratio at a respiratory rate of 18 breaths per minute, while the ventilator was set to produce 18 breaths per minute to maintain respiration if stimulation fails to trigger the ventilator. It delivered 4 cmH_2_O of pressure support to generate inspirations. Stimulation and ventilation were applied for 10 min while the diaphragm response was monitored to assess the ability to reliably produce diaphragm activation while maintaining respiration without interfering with ventilation.

### 2.4 Parameter optimization

The parameters required to reliably induce diaphragm activation with non‐invasive stimulation of the phrenic nerve were evaluated using an electrode array attached to a Digitimer DS8R stimulator as described. Respiratory responses to stimulation were measured as parameters were systematically varied and used to update the set of stimulation parameters required (Figure 2B). Electrode position, amplitude and pulse width, and stimulation frequency were systematically varied and their effects on diaphragm response were characterized (Figure 2C).

Respiratory responses to stimulation were recorded using pressure and flow sensors attached to a pitot tube section of an anesthetic circuit through which participants breathed freely, forming a seal with the lips around its end. Subjects maintained an open glottis and wore a nose clip to prevent the escape of air through the nose. The response of the diaphragm to stimulation was assessed by estimating the work done by the diaphragm following stimulation, providing a measure of the strength of the induced contraction. This was done by calculating the pressure‐volume work over the period following stimulation, i.e., ∫PdVdt.

The effect of electrode position was characterized by stimulating each cathode sequentially at a range of amplitudes using single stimulation pulses while the respiratory response was measured. This was performed with the head centered and then while looking to the left or to the right to assess the effect of head position on optimal electrode placement.

The electrode that induced a threshold response with the lowest stimulating current was selected. A diaphragmatic response to stimulation was defined as a deflection in the pressure and flow measures following stimulation with a magnitude of greater than three standard deviations from the mean for resting without stimulation. Responses were also visually inspected to manually confirm threshold measurements. The strength‐duration curve for the phrenic nerve was then characterized by measuring the threshold required to produce a measurable diaphragm response for a range of pulse widths using single stimulation pulses. The strength‐duration curve indicates the minimum stimulation current required to produce a response at a range of pulse widths. This provides important information on the responsiveness of the nerve to stimulation and the sensitivity of the nerve to changes in stimulation parameters. This provides valuable data for determining the most efficient stimulation parameters to produce reliable capture of the nerve as well as providing data on the variability of the parameters required to produce a response across a population.

The effect of stimulation frequency was investigated by delivering 200 ms pulse trains of varying frequency at the optimal electrode using 100 μs pulses at 125% of the threshold identified. The diaphragmatic pressure‐volume work produced by stimulation was calculated and integrated over the 1 s following stimulation to measure the effect of stimulation.

## 3 RESULTS

### 3.1 Participants

Ten participants (5 male, 5 female) were recruited for an initial assessment of the effect of non‐invasive phrenic nerve stimulation. Participants had a mean age of 31 (range 26–39) years and a mean weight of 77 (range 66–95) kilograms.

For a detailed assessment of stimulation parameters, 14 participants were recruited (7 male, 7 female). Participants had a mean age of 28 (range 19–50) years and a mean weight of 71 (range 52–98) kilograms.

### 3.2 Multi‐modal assessment

Bilateral non‐invasive phrenic nerve stimulation produced a diaphragm response, with the agreement of all multi‐modal measures, in all participants. Stimulation successfully produced a sufficient diaphragm response to trigger the ventilator in all participants, producing diaphragm exercise while ensuring consistent respiration during ventilation. The ventilator was driven by stimulation‐induced breaths alone for the full duration of stimulation in all participants, without any need to revert to ventilator‐triggered breaths.

**FIGURE 3:**
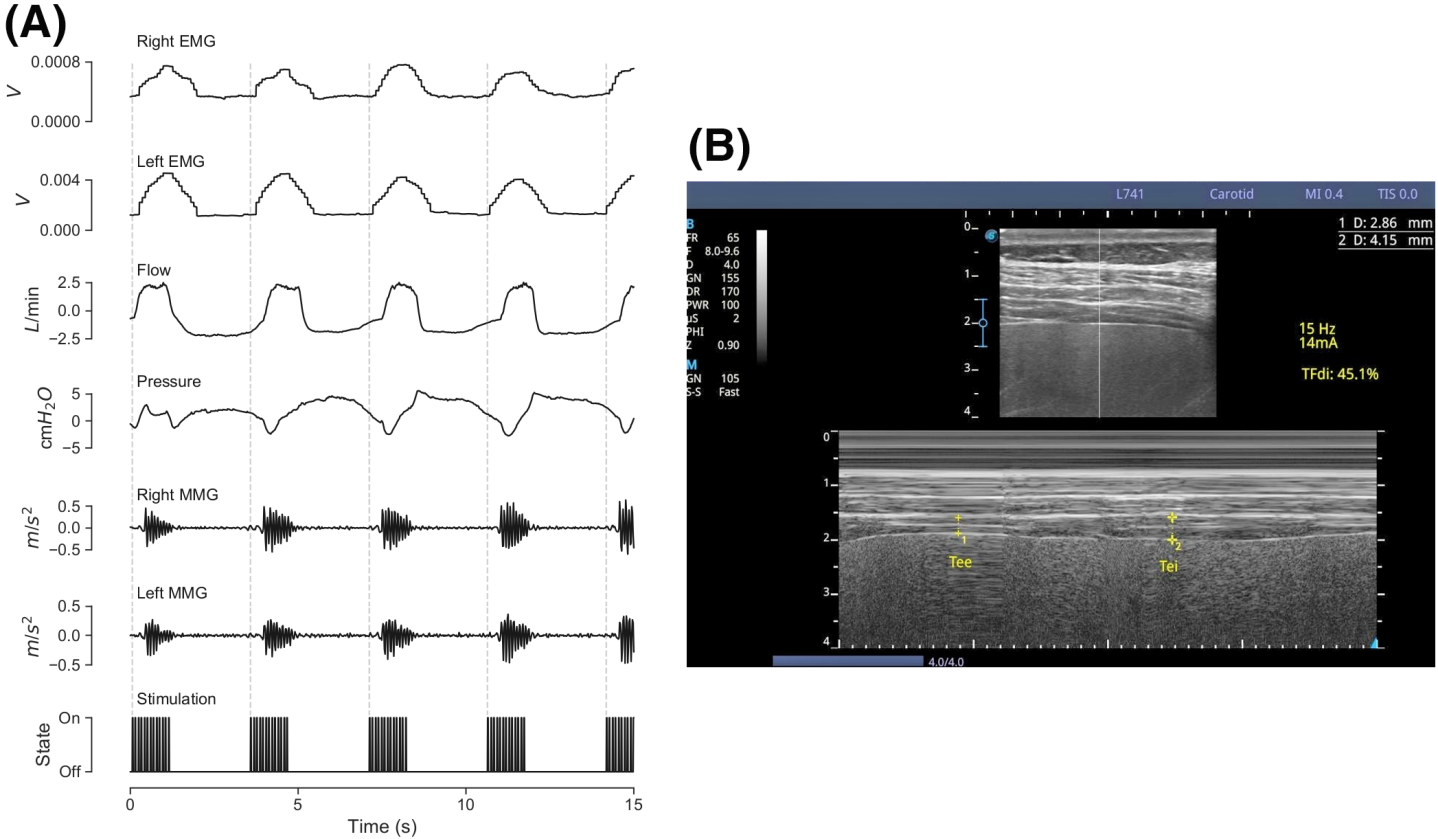
Response to phrenic nerve stimulation. (A) Bilateral EMG, respiratory flow and pressure, and bilateral MMG were recorded during stimulation. An example 15 s window during delivery of 20 mA, 200 μs pulses at 13 Hz with a pulse train duration of 1.1 s and an inter‐pulse interval of 2.2 s, synchronized to the ventilator, is shown. There is a clear response to phrenic nerve stimulation in all modalities measured. (B) Example diaphragmatic response to phrenic nerve stimulation assessed by ultrasound. Stimulation with 14 mA, 200 μs pulses at 15 Hz with a pulse duration of 1.1 s produced a diaphragm thickening fraction of 45.1%, demonstrating a robust diaphragm contraction in response to non‐invasive phrenic nerve stimulation

Figure 3 shows a representative 15‐s interval of multi‐modal recording during stimulation and ventilation. Electrical, mechanical, and respiratory measures all indicate significant diaphragm activation bilaterally in response to stimulation (Figure 3A). This is confirmed by ultrasonic visualization of diaphragm contraction during stimulation (Figure 3B). Non‐invasive phrenic nerve stimulation produces robust diaphragm contractions which can be detected using multiple measurement modalities.

The stimulation parameters used to produce consistent diaphragm responses in each participant are shown in Table S1. The mean stimulation amplitude was 23.9 ± 6.3 mA with a pulse width of 208.0 ± 38.2 μs. The mean within‐burst stimulation frequency was 13.4 ± 1.1 Hz.

No adverse reactions occurred during stimulation. All monitored vital signs remained within physiological ranges with no change during or following stimulation. Four participants reported discomfort during stimulation. This was described as a prickling sensation under the electrode. In each case, this appeared to be due to poor electrode adhesion and was successfully remedied by replacing the electrode that was inducing discomfort.

### 3.3 Electrode position

**FIGURE 4:**
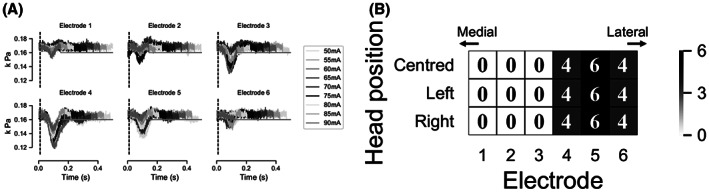
Effect of electrode position. (A) Example of differential pressure generated by single stimulation pulses at each electrode over a range of amplitudes. The response is sensitive to electrode positioning, with a rapid drop‐off in response between the optimal electrode and its neighbor. In this example, electrode 4 shows the strongest response with little response outside of this electrode. (B) Heatmap of optimal electrode position across participants; color indicates the number of participants for which that electrode was optimal. The number of participants is also shown within the heatmap. Electrode 1 is most medial with electrode 6 lateral. All participants responded to one of electrodes 4–6; the optimal electrode did not change with head position

The effect of variations in electrode position on the response to stimulation was evaluated using a custom linear array in a larger group of participants. In all participants, one cathode showed a greater response than all the others, with a rapid drop in diaphragm response following stimulation at the neighboring electrodes. An example of an electrode sweep over a range of amplitudes is shown in Figure 4A, where one electrode shows a clear response, while there is a smaller response at neighboring electrodes and little response at other electrodes.

The distributions of optimal electrodes across all participants for each head position are shown in Figure 4B. Most participants (6/14) had a maximal response at electrode 5 (68 mm from the midline), while 4 participants had a maximal response at the 4th (56 mm from the midline) and 6th (80 mm from the midline) electrodes. Notably, the optimal electrode did not vary with head position in any participant.

The optimal electrode for phrenic nerve stimulation could be reliably identified with a single sweep of single pulses at each electrode, allowing extremely rapid determination of the ideal electrode position for stimulation. Using single pulses at 2 Hz, the optimal electrode could be determined out of a set of six potential electrodes in 3 s during a single respiratory cycle. The optimal electrode for phrenic nerve capture also produced minor head movements on stimulation, likely due to activation of the underlying sternocleidomastoid muscle. In some participants, activation lateral to the optimal site for phrenic nerve activation produced movements of the arm and hand, likely due to activation of components of the brachial plexus lateral to the phrenic nerve. The locations for optimal activation of the phrenic nerve and activation of the brachial plexus did not overlap in any participants.

### 3.4 Strength‐duration curve

The strength‐duration relationship for stimulation parameters was characterized using the optimal electrode identified on assessment of electrode position. This demonstrates the minimum current required to produce nerve activation using a variety of pulse widths, characterizing the variability of parameters needed to produce a response and the sensitivity of responsiveness to changes in stimulation parameters. In all participants, diaphragm activation could be produced using reasonable stimulation parameters without excessive discomfort.

**FIGURE 5:**
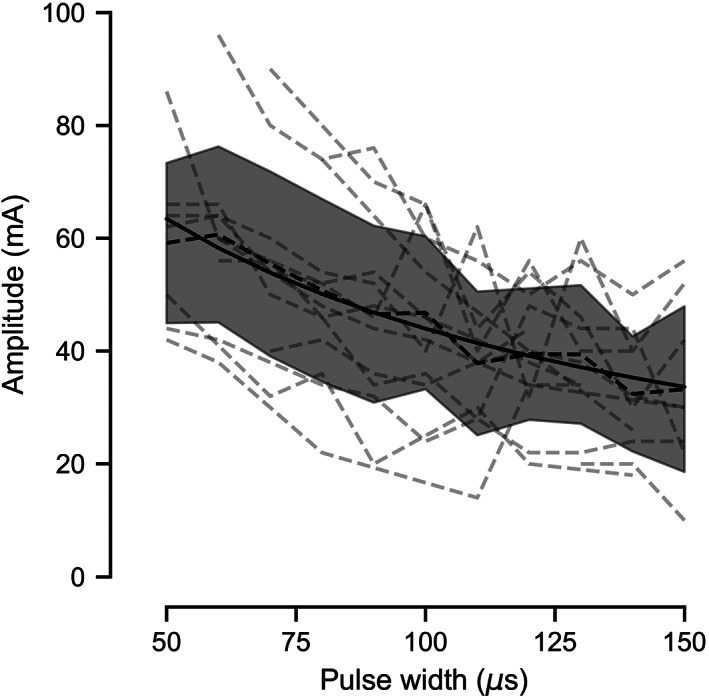
Strength‐duration curve showing the minimum stimulation current required to produce a diaphragm response for a range of pulse widths. The individual participants' strength‐duration curves are shown as gray dashed lines. The mean strength‐duration curve for all participants (*n* = 14) is shown as a black dashed line. The standard deviation is indicated by the shaded region. The amplitude required to induce diaphragm contraction decreases as pulse width increases. A hyperbola fit to the strength‐duration curves for all participants is shown as a solid line (*R* ^2^ = 0.94)

Threshold amplitudes were variable across individuals. There was a decrease in threshold as pulse width increased in all participants. The individual and mean strength‐duration curves for the phrenic nerve using single stimulation pulses are shown in Figure 5. A hyperbolic curve produced a good fit to the mean strength‐duration plot (*R* ^2^ = 0.94; mean absolute error = 1.95 mA), as is characteristic of strength‐duration relationships.

### 3.5 Stimulation frequency

Two hundred milliseconds of pulse trains of 100 μs pulses at 125% of the threshold identified on strength‐duration testing and at frequencies above 10 Hz produced a measurable diaphragm contraction in all participants with a nerve capture rate of 100%, i.e., delivery of a pulse train at the optimal electrode at 125% of the threshold amplitude always produced a robust diaphragm contraction.

**FIGURE 6:**
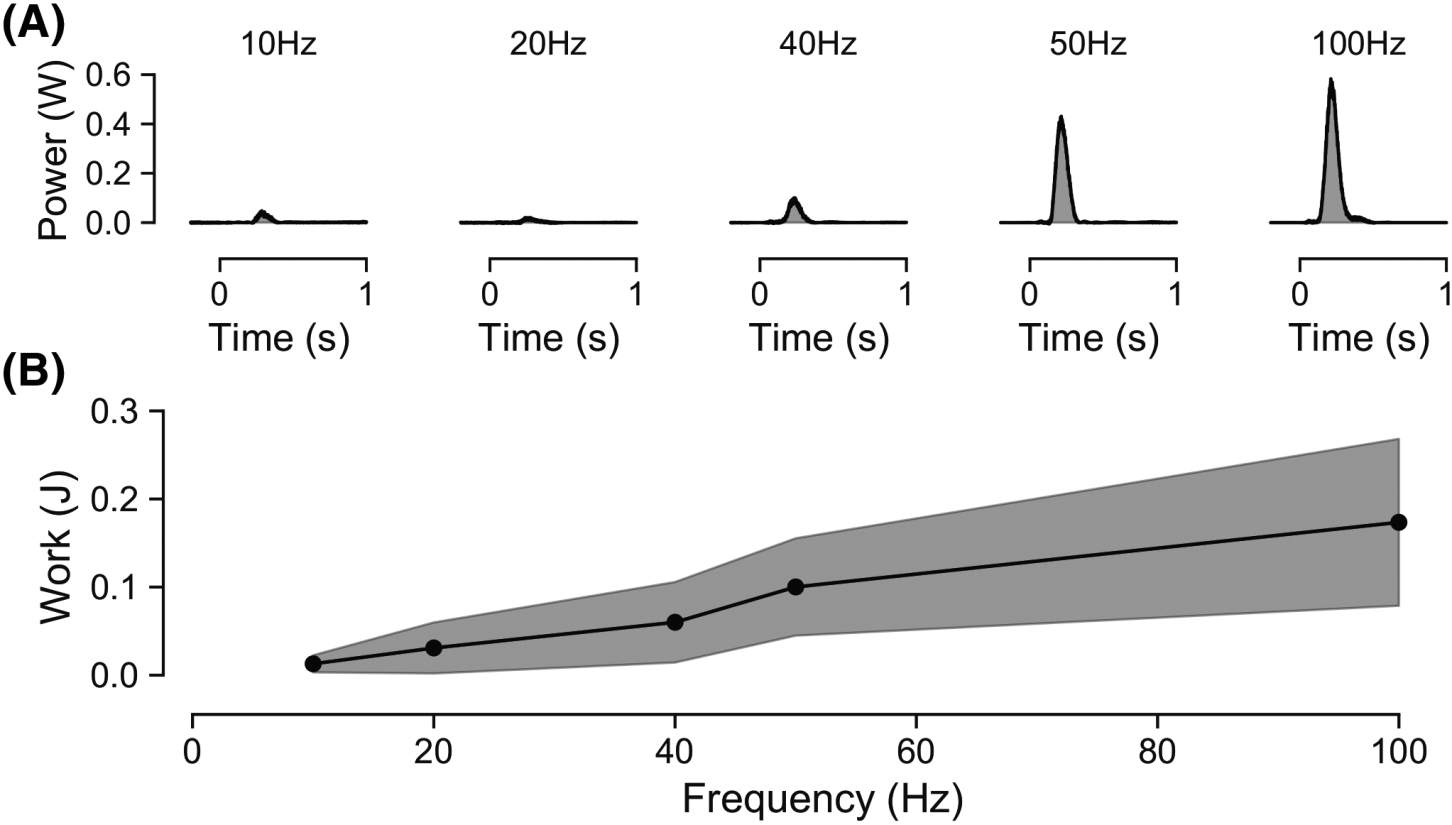
Effect of stimulation frequency on diaphragmatic work. (A) Example of diaphragm activity (W) following pulse train at a range of frequencies up to 100 Hz. (B) Diaphragmatic work done (J) following stimulation for a range of frequencies. Mean work done is shown. The standard deviation is shown as the shaded region. Increasing stimulation frequency induces greater diaphragmatic work

The work produced by stimulation increased as stimulation frequency increased. An example of the measured power (W) following pulse trains at varying frequencies is shown in Figure 6A. The total work (J) done during the induced contraction is shown in Figure 6B. This increases as stimulation frequency increases, with a sharp increase up to 50 Hz, and an average work at 100 Hz of 0.17 ± 0.09 J, corresponding to an inhaled volume of 609 ± 92 ml.

## 4 DISCUSSION

Our results indicate that non‐invasive stimulation of the phrenic nerve for inducing diaphragm contraction is feasible, and this does not require the application of pressure. We have characterized the stimulation parameters required for achieving reliable activation of the nerve.

Induction of diaphragm contraction using non‐invasive stimulation was confirmed using multiple modalities, including ultrasonic visualization. Further, this response was maintained during repeated stimulation and could reliably trigger the ventilator without the need to revert to ventilator‐triggered breaths and without interfering with respiration. These results provide strong evidence that non‐invasive stimulation can reliably produce robust diaphragm contractions suitable for maintenance of function during ventilation.

Monitoring of vital signs and clinical assessment revealed no adverse events during or following stimulation, suggesting that the phrenic nerve can be activated without producing side effects, such as unintended autonomic effects due to stimulation of the nearby vagus nerve or the carotid sinus higher in the neck. Some participants noted discomfort during stimulation due to the activation of cutaneous afferents and contraction of muscles in the neck. This discomfort was particularly notable at higher‐frequency stimulation. This discomfort during stimulation may limit the application of non‐invasive phrenic nerve stimulation to awake patients for prolonged periods but is unlikely to provide a major barrier to application in sedated, ventilated patients.

Activation of nearby structures, such as cutaneous afferents and neck muscles, is due to the lack of selectivity of surface electrodes. All structures under the electrodes are exposed to the electric field produced by stimulation, making targeted stimulation challenging. We found that the use of a concentric electrode arrangement was successful in constraining the area of activation, limiting off‐target effects, and allowing more targeted stimulation of the phrenic nerve. This was achieved by the enclosing anode limiting spread of the produced electric field beyond the area underlying the electrode. In order to allow greater control over the positioning of electrodes for assessment of sensitivity to electrode location and automatic identification of the optimal stimulation position, we further developed a linear electrode array with a surrounding anode. This allowed us to maintain targeted stimulation of the phrenic nerve with minimal off‐target effects while still retaining tight control over the precise location of stimulation.

The ability to activate the phrenic nerve non‐invasively is very sensitive to electrode position. Stimulation at the level of the cricoid cartilage reliably produced a response, but the lateral position required varied between individuals. All participants showed a response with stimulation using 1 cm cathodes centered between 56 mm and 80 mm from the midline, with most responding to stimulation centered at 68 mm, but the individual optimum position was variable. This has important implications for the practical application of this technique, as care must be taken to ensure electrodes are positioned appropriately.

However, our results also indicate that the optimal surface point for achieving nerve capture does not vary with head position. This is relevant for patients in intensive care, where prone positioning (i.e., lying the patient on their front) may be used to improve lung function. Prone positioning requires the head to be rotated up to 90° to the left or right; furthermore, it is generally used intermittently with periodic changes from supine (where the head will generally be in a neutral position) to prone and back again. Our results suggest that electrodes will not need to be repositioned when the head is turned. Further, our results demonstrate that it is possible to rapidly identify the optimal electrode position from an array using measurements of the diaphragm response; this raises the possibility of automatic electrode selection from a surface array to account for inter‐individual variability.

Our characterization of the strength‐duration relationship for non‐invasive stimulation of the phrenic nerve and the relationship between stimulation frequency and inhaled volume provide a demonstration of the ability to achieve meaningfully strong and powerful diaphragm contractions with non‐invasive stimulation with parameters that are achievable with standard stimulation hardware. This further provides a valuable resource for the development of non‐invasive stimulation systems by detailing the stimulation parameters required and their variability.

While our results were limited to the assessment of the use of pressure and flow measurements to assess the diaphragmatic response to stimulation, further work on other sensing modalities, such as lung sounds, may identify other methods of quantifying diaphragm activity and optimizing response to stimulation. Similarly, the results presented here used continuous mandatory ventilation to test interactions between stimulation‐induced breathing and ventilation systems by relying on the ability of stimulation‐induced breaths to trigger the ventilator. For application in an intensive care environment, it will also be valuable to implement a method to trigger the stimulator using the recorded respiratory signals in order to synchronize the stimulator with mandatory ventilation as an alternative to relying on assistive ventilation modes. Electrode location is also an important consideration for integration into existing intensive care workflows. Some ventilated patients will require central venous access. A linear array with the configuration presented here may provide a barrier to placing central venous catheters in the internal jugular vein. While using the subclavian vein for central access is a simple alternative, re‐designing the electrode array using the positioning data presented here may allow for reliable stimulation of the phrenic nerve without interfering with central venous access in the neck.

Further, while we can demonstrate reliable induction of diaphragm contraction in healthy volunteers, the ability to consistently produce sufficient diaphragmatic work to maintain function during mechanical ventilation in sedated, ventilated patients, whose body composition may vary over a broader range than our healthy volunteers, over the course of multiple days will need to be formally assessed. Similarly, the electrode impedances are likely to change over time and may require electrode replacement in situations where ventilation is required for longer than one week.^18^ A formal assessment of electrode performance over time will be required to determine the practical implications for prolonged use in intensive care units. However, issues with electrode replacement should be partially offset by easy landmark‐based electrode positioning and rapid identification of the optimal stimulating electrode, limiting the time and practical skills required to produce reliable phrenic nerve capture.

Overall, these results support the potential clinical validity of non‐invasive methods for maintaining diaphragm conditions and highlight the need for a clinical study of the efficacy of this approach in an intensive care environment. While care must be taken to ensure accurate electrode placement, the stimulation parameters required are well within the range of standard stimulation systems and well within safe limits. This opens the possibility for non‐invasive systems for maintaining diaphragm function in the intensive care setting, potentially improving ventilation outcomes while avoiding the risks and technical limitations of more invasive stimulation techniques.

## CONFLICT OF INTEREST

The authors declare no conflict of interest.

## AUTHOR CONTRIBUTIONS

*Conceived of and designed the studies*: Conor Keogh, Francisco Saavedra, Sebastian Dubo, Pablo Aqueveque, Paulina Ortega, Jonathan Jarvis, Brian Andrews, and James J. FitzGerald. *Designed and fabricated the equipment*: Adrian Poulton, Francisco Saavedra, Conor Keogh, Brian Andrews, Britam Gomez, Enrique Germany, Francisco Pastene, Daniela Pinto, and James J. FitzGerald. *Collected the data*: Conor Keogh, Francisco Saavedra, Brian Andrews, Sebastian Dubo, Britam Gomez, and James J. FitzGerald. *Performed the analysis*: Conor Keogh and Francisco Saavedra. *Wrote the manuscript*: Conor Keogh, Francisco Saavedra, Jonathan Jarvis, Brian Andrews, and James J. FitzGerald.*Supervised the project*: Pablo Aqueveque, Jonathan Jarvis, Brian Andrews, and James J. FitzGerald.

## Supporting information


Table S1

## References

[1] Levine S , Nguyen T , Taylor N , Friscia ME , Budak MT , Rothenberg P , Zhu J , Sachdeva R , Sonnad S , Kaiser LR , Rubinstein NA , Powers SK , Shrager JB Rapid disuse atrophy of diaphragm fibers in mechanically ventilated humans. N Engl J Med [Internet] 2008 Mar 27 [cited 2022 Jan 17];358(13):1327–35. Available from: https://www.nejm.org/doi/full/10.1056/nejmoa070447 18367735 10.1056/NEJMoa070447

[2] Dres M , Goligher EC , Dubé BP , Morawiec E , Dangers L , Reuter D , Mayaux J , Similowski T , Demoule A Diaphragm function and weaning from mechanical ventilation: an ultrasound and phrenic nerve stimulation clinical study. Ann Intensive Care [Internet] 2018 [cited 2022 Jan 17];8(1). Available from: https://pubmed.ncbi.nlm.nih.gov/29687276/ 10.1186/s13613-018-0401-y PMC5913054 29687276

[3] Dow DE , Cederna PS , Hassett CA , Kostrominova TY , Faulkner JA , Dennis RG . Number of contractions to maintain mass and force of a denervated rat muscle. Muscle Nerve [Internet]. 2004 Jul [cited 2022 Jan 28];30(1):77–86. Available from: https://pubmed.ncbi.nlm.nih.gov/15221882/ 15221882 10.1002/mus.20054

[4] Burgess LC , Venugopalan L , Badger J , Street T , Alon G , Jarvis JC , Wainwright TW , Everington T , Taylor P , Swain ID Effect of neuromuscular electrical stimulation on the recovery of people with COVID‐19 admitted to the intensive care unit: a narrative review. J Rehabil Med [Internet] 2021 Mar 1 [cited 2022 Jan 28];53(3). Available from: https://pubmed.ncbi.nlm.nih.gov/33634830/ 10.2340/16501977-2805 PMC8814855 33634830

[5] Onders RP . Functional electrical stimulation: restoration of respiratory function. Handb Clin Neurol [Internet] 2012 [cited 2022 Mar 3];109:275–82. Available from: https://pubmed.ncbi.nlm.nih.gov/23098719/ 23098719 10.1016/B978-0-444-52137-8.00017-6

[6] Dres M , Gama de Abreu M , Merdji H , Müller‐Redetzky H , Dellweg D , Randerath WJ , et al. Randomised clinical study of temporary transvenous phrenic nerve stimulation in difficult‐to‐wean patients. Am J Respir Crit Care Med [Internet]. 2022 Feb 2 [cited 2022 Mar 3]; Available from:. https://pubmed.ncbi.nlm.nih.gov/35108175/ 10.1164/rccm.202107-1709OC PMC9872796 35108175

[7] Dres M , On behalf of the RESCUE‐2 investigators group . Temporary transvenous diaphragm neurostimulation in mechanically ventilated patients: per protocol results from the RESCUE‐2 randomised controlled trial. Am Thorac Soc Int Conf Meet Abstr Am Thorac Soc Int Conf Meet Abstr [Internet]. 2021 May [cited 2022 Mar 3];A4668–A4668. Available from: www.atsjournals.org

[8] Onders RP , Elmo MJ , Kaplan C , Katirji B , Schilz R . Extended use of diaphragm pacing in patients with unilateral or bilateral diaphragm dysfunction: a new therapeutic option. Surgery [Internet]. 2014 Oct 1 [cited 2022 Mar 3];156(4):776–86. Available from:. https://pubmed.ncbi.nlm.nih.gov/25239317/ 25239317 10.1016/j.surg.2014.07.021

[9] Onders RP , Markowitz A , Ho VP , Hardacre J , Novitsky Y , Towe C , et al. Completed FDA feasibility trial of surgically placed temporary diaphragm pacing electrodes: A promising option to prevent and treat respiratory failure. Am J Surg [Internet]. 2018 Mar 1 [cited 2022 Mar 3;215(3):518–21. Available from:. https://pubmed.ncbi.nlm.nih.gov/29195690/ 29195690 10.1016/j.amjsurg.2017.10.054

[10] Reynolds SC , Meyyappan R , Thakkar V , Tran BD , Nolette MA , Sadarangani G , et al. Mitigation of ventilator‐induced diaphragm atrophy by transvenous phrenic nerve stimulation. Am J Respir Crit Care Med [Internet]. 2017 Feb 1 [cited 2022 Jan 17];195(3):339–48. Available from: https://pubmed.ncbi.nlm.nih.gov/27500981/ 27500981 10.1164/rccm.201502-0363OC

[11] Ayas NT , McCool FD , Gore R , Lieberman SL , Brown R . Prevention of human diaphragm atrophy with short periods of electrical stimulation. Am J Respir Crit Care Med [Internet]. 1999 [cited 2022 Jan 17];159(6):2018–20. Available from:. https://pubmed.ncbi.nlm.nih.gov/10351955/ 10351955 10.1164/ajrccm.159.6.9806147

[12] O'Rourke J , Soták M , Curley GF , Doolan A , Henlín T , Mullins G , et al. Initial assessment of the percutaneous electrical phrenic nerve stimulation system in patients on mechanical ventilation. Crit Care Med [Internet] 2020 [cited 2022 Jan 17];48(5):e362. Available from: /pmc/articles/PMC7161723/32191413 10.1097/CCM.0000000000004256 PMC7161723

[13] McGee DC , Gould MK . Preventing complications of central venous catheterization. N Engl J Med. [Internet]. 2009 Oct 7 [cited 2022 Jan 17];348(12):1123–33. Available from: https://www.nejm.org/doi/full/10.1056/nejmra011883 10.1056/NEJMra011883 12646670

[14] Severinghaus J . Electrophrenic respirator: description of a portable all‐electronic apparatus. Anesthesiology [Internet]. 1951 Jan 1 [cited 2022 Jan 17];12(1):123–7. Available from:. http://pubs.asahq.org/anesthesiology/article‐pdf/12/1/123/266501/0000542‐195101000‐00020.pdf 14799907

[15] Gandevia SC , McKenzie DK . Activation of the human diaphragm during maximal static efforts. J Physiol [Internet] 1985 Oct 1 [cited 2022 Jan 17];367(1):45. Available from: /pmc/articles/PMC1193052/?report=abstract 4057106 10.1113/jphysiol.1985.sp015813 PMC1193052

[16] Shaw RK , Glenn WWL , Hogan JF , Phelps ML . Electrophysiological evaluation of phrenic nerve function in candidates for diaphragm pacing. J Neurosurg [Internet]. 1980 Sep 1 [cited 2022 Jan 17];53(3):345–54. Available from:. https://thejns.org/view/journals/j‐neurosurg/53/3/article‐p345.xml 7420149 10.3171/jns.1980.53.3.0345

[17] Mier A , Brophy C , Moxham J , Green M . Repetitive stimulation of phrenic nerves in myasthenia gravis. Thorax [Internet] 1992 [cited 2022 Jan 17];47(8):640. Available from: /pmc/articles/PMC463928/?report=abstract 1329247 10.1136/thx.47.8.640 PMC463928

[18] Bîrlea SI , Breen PP , Corley GJ , Bîrlea NM , Quondamatteo F , Ólaighin G . Changes in the electrical properties of the electrode‐skin‐underlying tissue composite during a week‐long programme of neuromuscular electrical stimulation. Physiol Meas [Internet]. 2014 [cited 2022 Jan 17];35(2):231–52. Available from:. https://pubmed.ncbi.nlm.nih.gov/24434816/ 24434816 10.1088/0967-3334/35/2/231

