# Supplementary material for: Non‐invasive phrenic nerve stimulation to avoid ventilator‐induced diaphragm dysfunction in critical care
Source: Artif Organs. 2022 Apr 12;46(10):1988–97. doi: 10.1111/aor.14244 (PMC9790411; doi:10.1111/aor.14244)
Supplement: Supplementary file 1 — Table S1 [file AOR-46-1988-s001.docx]

**Table 1:** Electrical stimulation parameters used during initial functional testing

| Volunteer | Gender | Age | Frequency (Hz) | Pulse Width (us) | Right phrenic nerve amplitude (mA) | Left phrenic nerve amplitude (mA) |
| --- | --- | --- | --- | --- | --- | --- |
| 1 | F | 33 | 15 | 200 | 14 | 14 |
| 2 | M | 39 | 14 | 200 | 26 | 26 |
| 3 | F | 33 | 13 | 180 | 29 | 29 |
| 4 | F | 28 | 13 | 150 | 28 | 19 |
| 5 | F | 35 | 13 | 200 | 38 | 38 |
| 6 | M | 28 | 13 | 150 | 20 | 20 |
| 7 | M | 31 | 13 | 170 | 20 | 20 |
| 8 | F | 29 | 15 | 280 | 22 | 25 |
| 9 | M | 26 | 11 | 250 | 20 | 20 |
| 10 | M | 28 | 14 | 200 | 25 | 25 |
